# AGREEMENT BETWEEN PHYSICAL BEST AND FITNESSGRAM CRITERION-REFERENCED STANDARDS FOR MUSCULAR STRENGTH AND ENDURANCE

**DOI:** 10.1590/1984-0462/2021/39/2020018

**Published:** 2021-03-12

**Authors:** Gustavo Aires de Arruda, Diogo Henrique Constantino Coledam, Francys Paula Cantieri, Arli Ramos de Oliveira

**Affiliations:** a Universidade de Pernambuco, Recife, PE, Brazil.; b Instituto Federal de Educação, Ciência e Tecnologia de São Paulo, Boituva, SP, Brazil.; c Universidade Estadual de Londrina, Londrina, PR, Brazil.

**Keywords:** Physical fitness, Reference standards, Health, Child, Adolescent, Aptidão física, Padrões de referência, Saúde, Criança, Adolescente

## Abstract

**Objective:**

To verify the agreement between PHYSICAL BEST and FITNESSGRAM health-related criteria for muscle strength and endurance.

**Methods:**

This agreement study had a sample of 81 children and adolescents. Participants were submitted to the PHYSICAL BEST (Sit-up and Pull-up) and FITNESSGRAM (Curl-up and Modified Pull-up) test batteries. Additionally, FITNESSGRAM also proposed criteria for Pull-up test. Results of tests were classified in accordance with their respective criteria. Each group had an interval of seven days between the first and second battery of tests. Statistical analysis used the *Kappa* index (p<0.05).

**Results:**

Sit-up and Curl-up tests among the boys agreed in 72.2% (*Kappa*=0.368; p=0.004) of cases, and for the girls, in 64.4% (*Kappa*=0.130; p=0.076). Pull-up (PHYSICAL BEST *versus* FITNESSGRAM) agreed in 83.3% (*Kappa*=0.599; p<0.001) for boys. The agreement between Pull-up and Modified Pull-up (PHYSICAL BEST *versus* FITNESSGRAM) for boys was 47.2% (*Kappa*=0.071; p=0.533), and for girls, 44.5% (*Kappa*=0.102; p=0.120). The agreement between the Pull-up and Modified Pull-up tests (FITNESSGRAM) for boys was 58.4% (*Kappa*=0.215; p=0.143), and for girls, 44.5% (*Kappa*=0.102; p=0.120).

**Conclusions:**

For individual analysis over time, as well as for the comparison of passing rates between different populations, caution is advised when using different criterion-referenced standards for strength and endurance, particularly if using different tests.

## RESUMO

**Objetivo:**

Verificar a concordância entre os critérios relacionados à saúde para força e resistência muscular da PHYSICAL BEST e FITNESSGRAM.

**Métodos:**

Estudo de concordância com amostra composta de 81 crianças e adolescentes. Os participantes foram submetidos à bateria da PHYSICAL BEST (Abdominal e Flexão de cotovelos na barra) e da FITNESSGRAM (Abdominal modificado e Flexão de cotovelos na barra modificada). Adicionalmente, a FITNESSGRAM também propôs critérios para a Flexão de cotovelos na barra. Os resultados dos testes foram classificados de acordo com seus respectivos critérios. Houve um intervalo de sete dias entre as aplicações das baterias para cada grupo. A análise estatística utilizou o índice *Kappa* (p<0,05).

**Resultados:**

Os testes Abdominal e Abdominal modificado concordaram em 72,2% (*Kappa*=0,368; p=0,004) entre os meninos e 64,4% (*Kappa*=0,130; p=0,076) entre as meninas. Flexão de cotovelos na barra (PHYSICAL BEST *versus* FITNESSGRAM) concordou em 83,3% (*Kappa*=0,599; p<0,001) para os meninos. A concordância entre Flexão de cotovelos na barra e Flexão de cotovelos na barra modificada (PHYSICAL BEST *versus* FITNESSGRAM) foi de 47,2% (*Kappa*=0,071; p=0,533) para os meninos e 44,5% (*Kappa*=0,102; p=0,120) para as meninas. A concordância entre o teste de Flexão de cotovelos na barra e de Flexão de cotovelos na barra modificada (fitnessgram) foi de 58,4% (*Kappa*=0,215; p=0,143) para os meninos e 44,5% (*Kappa*=0,102; p=0,120) para as meninas.

**Conclusões:**

Para análises individuais ao longo do tempo, bem como para a comparação do atendimento entre diferentes populações, recomenda-se cautela ao usar diferentes critérios para a força e resistência, particularmente se forem usados testes diferentes.

## INTRODUCTION

Many investigations have been conducted to improve or develop health-related criterion-referenced (CR) standards for physical fitness.^1^ ^–^ ^3^ Physical fitness of children and adolescents has been largely examined in several studies.^4^ ^–^ ^6^ Health-related physical fitness batteries usually include tests for flexibility, muscular and cardiorespiratory fitness. The assumption for presence muscle strength and endurance tests is that these are important factors in carrying out daily activities and preventing injury, pain, and postural deviations.^7^ ^–^ ^9^ In addition, muscle strength and endurance are inversely correlated with body fatness,^10^ they can discriminate the nutritional status of children and adolescents,^11^ ^,^ ^12^ and their habitual physical activity^13^ and training status.^14^

PHYSICAL BEST^15^ and FITNESSGRAM^1^ ^,^ ^16^ ^,^ ^17^ CR standards has been widely used in physical fitness analysis, and different tests and CR standards are available. For abdominal muscles strength and endurance, Sit-up by PHYSICAL BEST^15^ and Curl-up by FITNESSGRAM1 have been recommended. For upper-body strength and endurance, PHYSICAL BEST developed CR standards for the Pull-up test,^15^ whereas FITNESSGRAM have CR standards for a variety of tests, such as the Pull-up and Modified Pull-up tests.^1^ ^,^ ^16^ ^,^ ^17^

Assuming that individuals who do not meet CR standards are at risk of having negative health outcomes, it is expected that the same individual would be classified in the same way when using different CR standards and tests. A previous study indicated that information obtained for flexibility using PHYSICAL BEST and FITNESSGRAM seems to be similar.^18^ However, information about the agreement between PHYSICAL BEST and FITNESSGRAM health-related CR standards for muscular strength and endurance are not available in the literature so far.

Such information may contribute to the choices of practitioners and researchers, and interpretation of CR standards and the analysis of study results that used different cut-off points. Thus, the purpose of the present study was to verify the agreement between PHYSICAL BEST and FITNESSGRAM health-related CR standards for muscle strength and endurance in children and adolescents.

## METHOD

This is an agreement study that was part of a larger project involving children and adolescents from Londrina City, Paraná State, Brazil. The larger project included information about physical activity, eating habits and consumption of alcoholic beverages, smoking, spinal pain, socioeconomic and demographic information from questionnaires. Subsequently, blood pressure, heart rate, and anthropometric measures were collected, and motor tests were performed. Study protocols were approved by the Ethics in Research Committee from the university where the study took place (Protocol No. 233/08).

Sample was composed by all children and adolescents from a sports program (Perobal Project). Participants were from the same school and lived in the western region of Londrina City, Paraná State, Brazil. The project was developed at the Physical Education and Sport Center of the Universidade Estadual de Londrina. Sample size was calculated according to the procedures described by Sim and Wright,^19^ considering *Kappa*'s value to be detected *Kappa*=0.60,^5^ value of null hypothesis for *Kappa*=0.0, and power=90%. The minimum number required to detect a statistically significant coefficient *Kappa* was 30 participants of each sex. Sample involved 81 subjects, 36 boys with a mean age of 12.8 (1.6) years old, and 45 girls with a mean age of 12.9 (1.5) years old.

Body mass was measured using a Plenna digital scale with 100 g scale, and height was measured using an stadiometer, with 0.1 cm scale, according to standard procedures.^20^ Finally, body mass index (BMI)=body mass (kg)/height (m)^2^ was calculated.

All participants were first submitted to PHYSICAL BEST^15^ battery, involving the Sit-up and Pull-up tests. Subsequently, FITNESSGRAM^1^ ^,^ ^16^ ^,^ ^17^ battery was performed, involving the Curl-up and Modified Pull-up tests. Results of the tests were classified in accordance with their respective CR standards. Additionally, given that FITNESSGRAM also has CR standards for the Pull-up, results of this test were classified by FITNESSGRAM standards. Each group had an interval of seven days between the first and second battery of tests. CR standards for the tests are presented in Chart 1.

**Chart 1:** Health-related criterion-referenced standards for physical fitness used in the study.

	PHYSICAL BEST				FITNESSGRAM					
	Pull-up (repetitions)*		Sit-up (repetitions)*		Pull-up (repetitions)†		Modified Pull-up (repetitions) ‡		Curl-up (repetitions) ‡	
Age	Boys	Girls	Boys	Girls	Boys	Girls	Boys	Girls	Boys	Girls
9	1	1	30	28	1	1	5	4	9	9
10	1	1	34	30	1	1	5	4	12	12
11	2	1	36	33	1	1	6	4	15	15
12	2	1	38	33	1	1	7	4	18	18
13	3	1	40	33	1	1	8	4	21	18
14	4	1	40	35	2	1	9	4	24	18
15	5	1	42	35	3	1	10	4	24	18
16	5	1	44	35	5	1	12	4	24	18

* AAHPERD;^15^

† FITNESSGRAM;^16^

‡ FITNESSGRAM.^1^ ^,^ ^16^ ^,^ ^17^

Shapiro-Wilk's test was used for normality analysis. Data that presented normal distribution was age, height, and Sit-up test in boys and girls. The body mass after transformation by Log10 presented normal distribution. The remaining variables presented non-normal distribution. The values of mean and standard deviation were used for variables with normal distribution. For variables with non-normal distribution, the median and percentiles 15.87 and 84.13 (range equivalent to a standard deviation) were presented. For comparisons between gender Student's “t” test for independent samples for variables with normal distribution and Mann-Whitney test for non-normal data were used. The agreement between health-related physical fitness CR standards classification was verified using the *Kappa* index, and its interpretation performed as suggested by Svanholm et al.:^21^ <0.20=Poor; 0.21 to 0.40=Regular; 0.41 to 0.60=Moderate; 0.61 to 0.80=Good; >0.80=Very good. Spearman correlation coefficient was used, and the interpretation was defined according to Tritschler:^22^ <0.30=Little or no correlation; 0.30 to 0.49=Weak; 0.50 to 0.69=Moderate; 0.70 to 0.89 Strong; >0.90=Very strong (positive and negative values were interpreted in the same way). A significance level of 5% was adopted.

## RESULTS

The characteristics of sample are presented in Table 1. Table 2 displays the agreement between Sit-up and Curl-up tests. In Tables 3 and 4, agreement values among CR standards for Pull-up and Modified Pull-up are shown.

**Table 1:** Sample characteristics according to gender*.

	Boys (n=36)	Girls (n=45)
Age (years old)	12.8 (1.6)	12.9 (1.5)
Body Mass (kg)	40.5 (31.0; 50.1)	44.1 (33.6; 58.0)
Height (cm)	151.7 (11.4)	151.5 (7.5)
BMI (kg/m^2^)† ‡	16.9 (15.1; 19.5)	18.9 (16.0; 24.2)
Sit-up (rep.)	39.3 (7.1)	35.0 (6.9)
Curl-up (rep.)†	39.5 (19.0; 80.0)	37.0 (22.36; 76.4)
Pull-up (rep.)†	0.5 (0.0; 3.0)	0.0 (0.0; 0.0)
Modified Pull-up (rep.)†	9.0 (3.0; 15.0)	5.0 (0.3; 8.0)

* mean and standard deviation were used for normal distribution variables, and median and percentiles 15.87 and 84.13 (range equivalent to a standard deviation) for non-normal data;

† p<0.05 difference between genders

‡ transformation by Log10 for comparison.

**Table 2:** 2×2 contingency of relative frequency for boys and girls classified for Sit-up (PHYSICAL BEST) and Curl-up tests (FITNESSGRAM).

		FITNESSGRAM — Curl-up		
		Pass (%)	Fail (%)	Total (%)
		Boys (n=36)		
PHYSICAL BEST – Sit-up	Pass	58.3	0.0	58.3
	Fail	27.8	13.9	41.7
	Total	86.1	13.9	100.0
		*Kappa*=0.368; p=0.004		
		Girls (n=45)		
PHYSICAL BEST – Sit-up	Pass	60.0	0.0	60.0
	Fail	35.6	4.4	40.0
	Total	95.6	4.4	100.0
		*Kappa*=0.130; p=0.076		

**Table 3:** 2×2 contingency of relative frequency for boys and girls classified for Pull-up test (PHYSICAL BEST and FITNESSGRAM).

		FITNESSGRAM — Pull-up		
		Pass (%)	Fail (%)	Total (%)
		Boys (n=36)		
PHYSICAL BEST – Pull-up	Pass	19.4	0.0	19.4
	Fail	16.7	63.9	80.6
	Total	36.1	63.9	100.0
		*Kappa*=0.599; p<0.001		
		Girls (n=45)*		
PHYSICAL BEST – Pull-up	Pass	8.9	0.0	8.9
	Fail	0.0	91.1	91.1
	Total	8.9	91.1	100.0

* *Kappa* index was not calculated because cut-off points are the same.

**Table 4:** 2×2 contingency of relative frequency for boys and girls classified for Modified Pull-up (FITNESSGRAM) and Pull-up tests (PHYSICAL BEST and FITNESSGRAM).

		FITNESSGRAM — Modified Pull-up		
		Pass (%)	Fail (%)	Total (%)
		Boys (n=36)		
PHYSICAL BEST – Pull-up	Pass	13.9	5.6	19.4
	Fail	47.2	33.3	80.6
	Total	61.1	38.9	100.0
		*Kappa*=0.071; p=0.533		
		Girls (n=45)		
PHYSICAL BEST – Pull-up	Pass	8.9	0.0	8.9
	Fail	55.6	35.6	91.1
	Total	64.4	35.6	100.0
		*Kappa*=0.102; p=0.120		
		Boys (n=36)		
FITNESSGRAM – Pull-up	Pass	27.8	8.3	36.1
	Fail	33.3	30.6	63.9
	Total	61.1	38.9	100.0
		*Kappa*=0.215; p=0.143		
		Girls (n=45)		
FITNESSGRAM – Pull-up	Pass	8.9	0.0	8.9
	Fail	55.6	35.6	91.1
	Total	64.4	35.6	100.0
		*Kappa*=0.102; p=0.120		

Variables that showed significant differences between sexes were BMI, with higher values for girls, and Curl-up, Pull-up, and Modified Pull-up, with higher values for boys. The Sit-up was the only test that did not indicate a significant difference between sexes (Table 1).

The correlations of tests used as indicators of trunk strength and endurance (Sit-up *versus* Curl-up) and for upper-body strength and endurance (Pull-up *versus* Modified Pull-up) were all positive, suggesting that the increase or decrease in performance in one test is accompanied by the same in the other test. However, this occurs with a moderate magnitude between Sit-up and Curl-up tests for boys (rho=0.644; p<0.001), and a weak magnitude for girls (rho=0.360; p=0.015). The correlation was weak for boys (rho=0.442; p=0.007) and girls (rho=0.371; p=0.012) between Pull-up and Modified Pull-up tests.

Table 2 indicates the agreement between PHYSICAL BEST and FITNESSGRAM CR standards for Sit-up and Curl-up tests. Among boys, a regular agreement was found, with CR standards agreeing in 72.2% of cases. Among girls, there was a poor agreement, with 64.4% of cases agreeing between classifications.

Table 3 presents the agreement for Pull-up classifications proposed for PHYSICAL BEST and FITNESSGRAM. For boys, CR standards agreed in 83.3% of cases, and the agreement between classifications was moderate. For girls, cut-off points are the same, and Table 3 indicates the passing rates CR standards. Results indicated that only 8.9% of girls passed CR standards.

The agreement between Modified Pull-up (FITNESSGRAM) and Pull-up (PHYSICAL BEST) tests classification for boys was 47.2%, the agreement for girls was 44.5%, both classified as poor. The agreement between FITNESSGRAM CR standards for Modified Pull-up and Pull-up tests for boys was regular, with 58.4% of cases classified in the same way. Among girls, the agreement was 44.5%, classified as poor by the *Kappa* index (Table 4).

## DISCUSSION

The main results of this study were that the agreement between CR standards for Sit-up and Curl-up tests was regular for boys and poor for girls. For the Pull-up test (PHYSICAL BEST *versus* FITNESSGRAM) there was a moderate agreement between CR standards for boys (for girls, the agreement was not analyzed because the cut-off points are the same). The agreement between Pull-up (PHYSICAL BEST) and Modified Pull-up (FITNESSGRAM) tests was poor for boys and girls. For Pull-up and Modified Pull-up tests, with CR standards proposed by FITNESSGRAM, the agreement was regular and poor for boys and girls, respectively.

Abdominal muscle strength and endurance tests performed by trunk flexion are widely used because low scores in these tests indicate a risk factor for the emergence of low back pain.^9^ In addition, this component is inversely correlated with health risk factors, such as elevated waist circumference and BMI,^10^ and seems to discriminate trained and non-trained adolescents.^14^ Similarly, upper-body strength is inversely associated to being overweight,^12^ and differ significantly according to physical activity levels13 and nutritional status.^11^

The Sit-up, Curl-up, Pull-up, and Modified Pull-up tests aim to evaluate the same component of health-related physical fitness, muscle strength, and endurance. However, the correlation between the scores for the Sit-up and Curl-up tests was moderate for boys and weak for girls. With respect to Pull-up and Modified Pull-up tests, the correlation was weak for boys and girls. Higher coefficients were expected for tests that aim to assess the same characteristics.

Moderate and weak levels of correlations between the field tests could indicate that the different tests are measuring different factors; on the other hand, this does not appear to be the case. Pate et al.,^23^ verified correlations from 0.24 to 0.47 between the Pull-up and Modified Pull-up, for girls and boys, respectively, but results of the principal components analyses of performances for field tests load significantly on the same factor, suggesting that the tests measure the same construct.

Another relevant aspect was that the strength and endurance performance of the trunk region was different between boys and girls only for the Curl-up. This fact suggests that the differences between sexes can be influenced by the test used. This was unexpected given that both Sit-up and Curl-up tests assess the same component, which should have produced similar results. Similarly, CR standards for the same component should result in the same classification (Pass *versus* Fail), once they are considering the assessment of the same body region. This fact was not verified in the present study for tests used as indicators of strength and endurance of the abdominal region, because the agreement presented by Sit-up and Curl-up was regular for boys and poor for girls.

No research that investigates the agreement between PHYSICAL BEST and FITNESSGRAM CR standards for muscle strength and endurance were conducted until now. Available information is related to the agreement between CR standards of the FITNESSGRAM Push-up, and alternative tests of upper-body strength and endurance tests.^4^ ^,^ ^5^

A study developed with children aged 8–11 years old verified the agreement of FITNESSGRAM CR standards for Push-up and Modified Pull-up. Agreement ranged from moderate to good (*Kappa*=0.48 to 0.72) for boys, which is considered acceptable. For girls, all values were classified as poor (*Kappa*= −0.04 to 0.18), which is considered unacceptable. Approximately 20% of boys were classified differently between tests at each age level, and most misclassified boys passed the Modified Pull-up standard, but failed the Push-up; for girls, over 40% in each group were classified differently between tests. Researchers concluded that practitioners should not be encouraged to use the Push-up and Modified Pull-up tests interchangeably.^5^

In the present study, similar results were found for girls, for the agreement between Modified Pull-up (FITNESSGRAM) and Pull-up (PHYSICAL BEST), in which 55.6% were classified differently. Of these, all girls passed the Modified Pull-up CR standards, but failed the Pull-up. Low values were found for boys: 52.8% were misclassified; most passed the Modified Pull-up standard, but failed the Pull-up (47.2%). For the Modified Pull-up and Pull-up with FITNESSGRAM CR standards, the disagreement was 41.6%; of them, 33.3% passed the Modified Pull-up and failed the Pull-up.

One problem that suggested with the Pull-up test is that body weight can affect the score. A participant must overcome his or her entire body weight to perform each repetition, which hampers the performance of heavier individuals.^24^ Another important fact is that this field test cannot detect individual differences among students with lower levels of muscular strength and endurance.^24^ ^,^ ^25^

In the first National Children and Youth Fitness Study,^26^ between 10 and 30% of boys aged 10 to 14, and more than 60% of girls aged 10 to 18 had zero scores on the Pull-up test. This problem seems be attenuated with the use of the Modified Pull-up test. This is evidenced by the values of subjects who achieved CR standards for Modified Pull-up in the present study and the fact that the Modified Pull-up presented higher values of repetitions than the Pull-up test, in which the mean number of repetitions was less than one, thus explaining in part the low agreement between the two tests.

The absolute overload imposed on the Modified Pull-up seems to be diminished compared to the Pull-up because of the positioning in the Modified Pull-up, allowing partial contact of the body with the ground, whereas body weight is supported in its entirety in the Pull-up. Scores for both tests seem to be influenced by factors like body weight and body fat percentage,^25^ ^,^ ^27^ ^,^ ^28^ a negative correlation between the Modified Pull-up and body fat was found for boys, with rho=-0.51. For girls, the value obtained was rho=-0.52.^28^ Magnitude of the overload generated by factors such as body fat seems to be quite similar for both tests.^27^ ^,^ ^28^

Sherman and Barfield^5^ found that the agreement of CR standards for FITNESSGRAM Push-up and Pull-up are affected by factors like age and gender. For girls from 8 to 10 years old, moderate to good agreement was found (*Kappa*=0.44 to 0.64). As to 11 year-old girls, regular agreement (*Kappa*=0.34) with a percentage agreement of 0.67 (n=43) was observed; for boys, the same age agreement was a little higher, but also regular (*Kappa*=0.40); and the percentage agreement was very similar at 0.70 (n=44). Boys from 8 to 9 years old had regular agreement (*Kappa*=0.22 to 0.24). As to 10 year-old boys, the agreement was moderate (*Kappa*=0.48). The authors discussed, based on previous studies^23^ ^,^ ^25^ that lack of agreement could also be due to the varying muscle groups emphasized by each test. The Push-up, for example, emphasizes pectoralis major and triceps, whereas the Pull-up emphasizes the latissimus dorsi and biceps.

Similar results to those by Sherman and Barfield^5^ were obtained in another study, in which the agreement values ranged from regular to moderate for Push-up, and Modified Pull-up for both boys and girls.^4^ These results corroborate those indicated in the present study, concerning the agreement between Pull-up (PHYSICAL BEST) *versus* Modified Pull-up (FITNESSGRAM), and Pull-up *versus* Modified Pull-up (FITNESSGRAM).

Unlike in prior studies,^4^ ^,^ ^5^ the agreement between CR standards established by PHYSICAL BEST and FITNESSGRAM for Pull-up and Modified Pull-up was analyzed in the present study, tests which assess similar muscle groups. Results indicate that the agreement is dependent on the test used. When analyzing the agreement of PHYSICAL BEST and FITNESSGRAM CR standards for the same test (Pull-up), the agreement was classified as moderate for boys. However, when Pull-up (PHYSICAL BEST) and Modified Pull-up (FITNESSGRAM) tests were analyzed, the agreement was poor for boys and girls. The agreement between FITNESSGRAM CR standards for Pull-up and Modified Pull-up tests was classified as regular for boys, and poor for girls.

The Sit-up and Curl-up tests were used as indicators of trunk strength and endurance. Results of this study suggest that the agreement between CR standards was unacceptable. Possible aspects that affect the tests results are differences in protocol for tests. Cadence-based Curl-up test is recommended in FITNESSGRAM, whereas Sit-up is recommended in PHYSICAL BEST, which is cadence free. Curl-ups are intended to use different muscles over a more restricted range of motion than Sit-ups.^29^ Different forms of Sit-ups seem to activate the hip flexor muscles more than Curl-ups. Curl-up seems to activate the external obliques, internal obliques, and transverse abdominis more than other kind of Sit-up.^30^ Similar studies about CR standards agreement between these tests were not found.

Results of the present study suggest that the agreement for Pull-up and Modified Pull-up was unacceptable. The results indicate that, when performing the analysis of health-related physical fitness over time, only one test and CR standards are recommended to be used. In addition, one should be careful when comparing the results of studies that verified the passing rates with different CR standards for health-related physical fitness for muscular strength and endurance, except for the Pull-up test, using PHYSICAL BEST or FITNESSGRAM CR standards.

The disagreements between CR standards seem to occur due to the different tests and cut-off points. When dealing with motor performance testing, there is not a gold standard test like there is in some physiological measures. This is a limiting factor in the study, because the investigator could verify which criterion is most suitable for use if there were a gold standard measure. Moreover, the agreement between was not described according to the age group, and yet this does seem to be influenced by age.^5^ Thus, future studies should check the agreement of strength and endurance motor tests CR standards within different age groups, as well as the possible influence of biological maturity, due to the scarcity of information when compared to other components of physical fitness, such as cardiorespiratory fitness and body composition. Another important aspect to be investigated is the performance of CR standards in indicating outcomes, such as the presence of back pain and postural deviations.

In conclusion, poor to regular agreement between CR standards for Sit-up (PHYSICAL BEST) and Curl-up (FITNESSGRAM) was found. The only test used for both the proposed CR standards (PHYSICAL BEST and FITNESSGRAM) was Pull-up, and the agreement for boys between CR standards for this test was moderate. When subjects were classified into CR standards for different upper-body strength and endurance tests, the agreement was regular for boys, and poor for girls, indicating that the agreement in the classification CR standards for boys seems to reduce mainly when different tests are used to assess the same component.

Practitioners should not be encouraged to use FITNESSGRAM and PHYSICAL BEST health-related CR standards for strength and endurance interchangeably. For analysis over time as well as to the comparison of passing rates between different populations, caution is advised when using different CR standards for strength and endurance, especially if using different tests.
